# Role of the Microenvironment in Mesenchymal Stem Cell-Based Strategies for Treating Human Liver Diseases

**DOI:** 10.1155/2021/5513309

**Published:** 2021-11-16

**Authors:** Shan-jing Xu, Li-ping Ye, Wei Wang, Ya-hong Chen, Jian Dong, Xin-li Mao, Shao-wei Li

**Affiliations:** ^1^Shaoxing University School of Medicine, Shaoxing, Zhejiang, China; ^2^Key Laboratory of Minimally Invasive Techniques & Rapid Rehabilitation of Digestive System Tumor of Zhejiang Province, Taizhou Hospital Affiliated to Wenzhou Medical University, Linhai, Zhejiang, China; ^3^Department of Gastroenterology, Taizhou Hospital of Zhejiang Province Affiliated to Wenzhou Medical University, Linhai, Zhejiang, China; ^4^Institute of Digestive Disease, Taizhou Hospital of Zhejiang Province Affiliated to Wenzhou Medical University, Linhai, Zhejiang, China; ^5^Taizhou Hospital of Zhejiang Province Affiliated to Wenzhou Medical University, Linhai, Zhejiang, China; ^6^Health Management Center, Taizhou Hospital of Zhejiang Province Affiliated to Wenzhou Medical University, Linhai, Zhejiang, China; ^7^Tianjin Key Laboratory of Information Sensing & Intelligent Control, Tianjin University of Technology and Education, Tianjin, China

## Abstract

Liver disease is a severe health problem that endangers human health worldwide. Mesenchymal stem cell (MSC) therapy is a novel treatment for patients with different liver diseases due to its vast expansion potential and distinctive immunomodulatory properties. Despite several preclinical trials having confirmed the considerable efficacy of MSC therapy in liver diseases, the questionable safety and efficacy still limit its application. As a precursor cell, MSCs can adjust their characteristics in response to the surrounding microenvironment. The microenvironment provides physical and chemical factors essential for stem cell survival, proliferation, and differentiation. However, the mechanisms are still not completely understood. We, therefore, summarized the mechanisms underlying the MSC immune response, especially the interaction between MSCs and the liver microenvironment, discussing how to achieve better therapeutic effects.

## 1. Introduction

As the largest internal organ, the liver accepts a dual blood supply from both the portal vein and hepatic artery, causing hepatocytes to be constantly exposed to metabolites and toxins from the gastrointestinal tract and peripheral circulation [1]. Liver injury initiated by viruses, alcohol, drug abuse, and other physical and chemical factors is very widespread in clinical practice [2, 3]. All of these agents can activate the inflammatory response without timely medical intervention, subsequently resulting in the development of hepatic inflammation, liver failure (HF), cirrhosis, and hepatocellular carcinoma (HCC), which account for 3.5% of deaths around the world [3, 4].

It is generally acknowledged that the liver has an extraordinary regenerative capacity. Many animal experiments and clinical studies have found that following simple partial hepatectomy (PH), the residual liver can rebuild to its initial mass within just seven days to a few weeks in rodents and humans. Furthermore, even after the surgical removal of up to 70% of the liver, the mass can regenerate to its primary state without functional loss [5]. The fundamental driving force is the integrity of the liver segment and the compensatory hypertrophy and hyperplasia of the resident cells.

However, during the regeneration of the persistent chronically or severely injured liver, constant stimulation may induce hepatocyte apoptosis, recruitment of inflammatory cells, activation of hepatic stellate cells (HSCs), and formation of liver fibrosis.

When the compensation capacity of hepatocytes is insufficient for recovery from injury, resident liver progenitor cells (LPCs) and some specific hepatic cells are activated, subsequently reprogramming and participating in liver regeneration by differentiating into hepatocytes [6]. Once the balance between destruction and regeneration has been broken, fibrosis is inevitable, and the liver function progressively deteriorates. Liver failure usually occurs after roughly 80%-90% of the parenchyma has been destroyed.

Liver transplantation (LT) is regarded as an efficient method for resolving end-stage chronic liver disease or exacerbating acute liver failure. Still, its clinical application is markedly restricted by the scarcity of donor sources, costliness, risk of graft-versus-host disease (GvHD), and a high rejection rate, among other issues [5–8].

The rapid development of emerging regenerative medicine has provided new options [9]. Based on the inherent capacity of the liver, allogeneic hepatocyte transplantation at one time has been regarded as an alternative approach to transplantation. However, only a modest benefit was reported in limited data, as well as a series of problems with low availability, including failed donor implantation, a high rejection rate, and low viability in culture [10, 11].

According to recent studies, a series of experiments using different origin-derived extrahepatic stem cells have shown therapeutic potential for liver function improvements, such as embryonic stem cells (ESCs), induced pluripotent stem cells (iPSCs), hematopoietic stem cells (HSCs), and mesenchymal stem cells (MSCs) [12–14]. Among these origin cells, MSCs are particularly attractive due to their multisource supply, low immunogenic profile, the capability of self-renewal, and multidirectional differentiation. They thus have rapidly become the focus of liver regenerative medicine [15].

Several preclinical trials had been designed to explore the potential utility of MSCs for different liver diseases (such as hepatitis, fibrosis, liver failure, and primary biliary cirrhosis (PBC)), and some of them have generated cheering results [16, 17].

While most MSC applications in liver disease have demonstrated protective effects, Baertschiger et al. found that MSCs expressed *α*-SMA, which merged with collagen deposition in an acute liver injury (ALI) model generated by 2/3 PH, suggesting that MSCs can adopt a fibrogenic phenotype. Furthermore, Mohamadnejad et al. found that bone marrow-derived mesenchymal stem cell (BM-MSC) transplantation through a peripheral route had no effect on decompensated cirrhosis for patients. Other reports confirmed this conclusion. Some have hypothesized that infused MSCs may differentiate into fibroblast-like cells, causing further fibrosis [18, 19].

This review is aimed at providing an update on the interaction between MSCs and the liver microenvironment and discussing the potential perspectives of MSC for liver damage.

## 2. Definition of MSCs

MSCs, also known as multipotent mesenchymal stromal cells, are nonhematopoietic fibroblast-like cells that can be isolated from most tissue and are generally found in perivascular niches [20]. Adult BM-MSCs are by far the universal source of human MSCs [15, 21]. Even when the sources are different, MSCs retain many similar properties *in vitro*.

In 2006, the International Society for Cellular Therapy (ISCT) committee defined MSCs as cells that (1) show adhesion to plastic *in vitro* under standard culture conditions, (2) show the expression of specific surface antigens, and (3) have a multidirectional differentiation ability [22].

Aside from their ability to self-replicate as well as their multilineage differentiation capacity, similar to true stem cells, MSCs also have the following advantages: (1) MSCs are widely distributed and can be obtained easily, (2) their low immunogenicity makes allogeneic transplantation possible, (3) they are capable of immunomodulation, and (4) they can also produce secretomes to favor regeneration and injury repair. These benefits make MSCs the most promising cells nowadays for various diseases [19, 23].

Since the first clinical trial using MSCs, over 10,000 patients have been treated for various diseases with allogeneic or autologous MSCs, including GvHD, ischemic heart disease, stroke, Crohn's disease, and amyotrophic lateral sclerosis (ALS), and most of them have got not bad results [16, 17, 24].

For example, Wang et al. found that allogeneic MSC infusion could improve systemic inflammation and promote the recovery of liver inflammation in PBC mice after six weeks' consecutive intervention. A subsequent pilot study of MSC fusion in PBC patients further verified this result, finding that MSC treatment could ease the symptoms of patients who were unresponsive to traditional therapy as well as improve the Child-Pugh and model for end-stage liver disease scores and performance status [25, 26]. Analogous results have been confirmed for other liver diseases [27–30].

## 3. The Structure of the Liver

**Figure 1:**
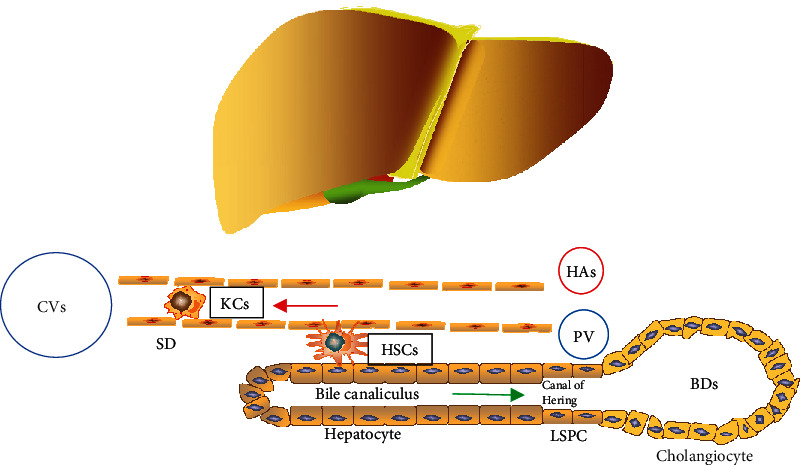
The structure of liver tissue. Liver tissue accepts a dual blood supply from the portal vein (PV) and the hepatic artery (HA). Hepatocytes produce the bile, which flows into the bile ducts (BDs). Hepatic arterioles, terminal branches of the portal vein, and the most minor bile ducts form the compact portal tracts. Bile ductules drained into the bile ducts via the canals of Hering, which contain LSPCs. The narrow space between trabeculae and the endothelium of blood sinusoids is called the spaces of Disse (SD), which participate in the bidirectional traffic between blood and hepatocytes. In the case of acute injury or chronic liver diseases, hepatic progenitor cell activation occurs in response to different inflammatory cytokines. CVs: central veins; LSPCs: liver stem/progenitor cells; KCs: Kupffer cells; HSCs: hepatic stellate cells.

The liver has a high degree of vascularization, and the permeable fenestrated endothelia have a slow blood flow within, providing an excellent environment in which hepatic cells can thrive and communicate [1, 31]. The structure presented in Figure 1 is the basic structure of the liver. Hepatocytes are arranged in two rows, forming the trabeculae. Spaces between the hepatocyte rows include the biliary canal, evacuating bile to the bile ductule (BDL) through the channel of Hering (CH). Between the trabeculae lies structures called blood sinusoids (BSs), formed by LSECs and lined with Kupffer cells (KCs). Between the trabeculae and blood sinusoids is a narrow space called the spaces of Disse (SDs), containing stellate cells, which participate in the bidirectional traffic of hepatocytes with blood. The liver also has other immune cells, such as resident natural killer (NK) and dendritic cells [5, 32].

The functional units of the liver are the lobules, which are composed of trabeculae and BSs arranged regularly. The edges of the three lobules meet to form portal areas, which contain a portal vein (PV), hepatic artery (HA), and bile duct (BD). Branches of blood vessels merge to form sinusoids, and the blood from the HAs and PVs mixes before being imported into the central vein (CVs), hepatic vein, and inferior vena cava progressively [32].

The bile flows in the opposite direction as the blood and drains into the BD. The small HAs, the terminal branches of the PV, and the smallest BD form a compact structure called the portal tract. Liver trabeculae are imported into the BDLs via CHs containing liver stem/progenitor cells (LSPCs) [33, 34].

Besides, the liver comprises parenchymal cells, such as hepatocytes, cholangiocytes, and LSPCs, as well as nonparenchymal cells, such as HSCs, LSECs, KCs, and other immune cells, both of which can present antigens as well as secrete cytokine and chemokine, thereby initiating immune responses [35–37].

### 3.1. The Microenvironment of the Liver

The liver microenvironment is composed of cells, extracellular matrix, cytokines, and nutrition. All of them are in the dynamic changes *in vivo*. The liver microenvironment is a multidirectional interaction complex (cell-matrix-cell) that plays an important role in the maintenance of normal functions [5]. For example, hepatocytes act as polarized cells, owning three different plasma membrane domains: the basolateral domain (contacts SDs), the lateral domain (tight junctions join adjacent liver cells), and the apical domain (secretes bile).

### 3.2. The Interaction between MSCs and the Liver Microenvironment

Growing evidence has confirmed that the behavior of MSCs is closely associated with surrounding microenvironments. MSC-based cell therapy requires the isolation of stem cells from their native donor microenvironment, culture in vitro in a certain microenvironment, and then transplantation of the cells and/or their production into the recipient microenvironment, participating in liver regeneration. The microenvironment provides physical and chemical factors essential for stem cell survival, proliferation, and differentiation. The donor microenvironment, including the age and physical state of the donor, affects the viability, differentiation potential, and regenerative capacity of MSCs. For example, François et al. had observed that the MSC suppressive function is closely connected to the magnitude of IDO response of individual donors [38]. The experiment had demonstrated that MSCs derived from the donors with low IDO respond remarkedly less effectively than cells derived from the donors with high responses. However, some studies have confirmed that culturing with IFN-*γ* and TNF-*α* in vitro does activate the IFN-*γ* and NF-*κ*B signaling pathways of MSCs, which are harvested from healthy volunteer donors, normalize the weak and strong lines, and enhance the immunosuppressive function both in vitro and in vivo [39]. Besides, the markers are remarkably uniform in their expression among different individual donors.

In addition, the culture microenvironment in vitro, also called the “synthetic niche,” also strongly influences the abilities of extrinsic MSCs. In general, the synthetic niche consists of a complex set of mechanical, biophysical, and biochemical factors, which influence the behavior of MSCs by imitating the structure of microenvironment structure in vivo and providing the scaffold for cell adhesion, thereby helping to improve the scale and efficiency of stem cell culture in vivo and promote stem cell differentiation [40, 41]. Furthermore, the recipient microenvironment also plays a prominent role in MSC-based liver regeneration, which relies on not only the interaction between exogenous transplanted cells and recipient microenvironment but also, at least in part, the secretome (e.g., direct protein secretions and extracellular vehicles (EVs), including exosomes and macrovesicles) of MSCs exerting a similar therapeutic potential, which may form the basis of future MSC therapies.

But several studies have found that although MSCs initially target sites of injury and differentiate into multiple cell types, the rate of engraftment following systemic administration of MSCs is very low. Therefore, the more accepted explanation is that MSCs exert their therapeutic potential through a paracrine effect, modulating immune responses and homing into the injury site. Substantial research has shown that MSCs can release multiple bioactive factors (i.e., cytokines, chemokines, growth factors, and EVs) that play an important role in a cell's fate. However, the fate of MSCs themselves is largely influenced by the inner microenvironment.

**Figure 2:**
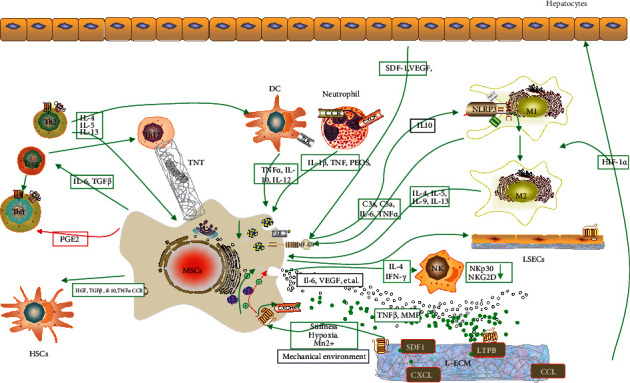
The crosstalk between mesenchymal stem cells (MSCs) and liver microenvironment. (1) MSCs regulate the survival, migration, differentiation proliferation, and apoptosis of liver cells and immune cells to cobuild the microenvironment promoting liver regeneration via cell-cell interactions and paracrine and signal pathways. (2) The capacity of immune regulation of MSCs might be influenced by the physical and chemical properties of ECMs, cytokines, cell-cell interaction, and signal pathways. HSCs: hepatic stellate cells; Tregs: regulatory T cells; Th: T helper; LSECs: liver sinusoid epithelial cells; MMP: matrix metalloproteinase; DCs: dendritic cells; M: macrophages; NK cells: natural killer cells; LTBPs: latent-TGF-*β*-binding proteins; L-ECM: liver-extracellular matrix; SDF-1: stromal cell-derived factor 1; HIF-1a: hypoxia-inducible factor-1a; TGF: transforming growth factor-beta; PGE2: prostaglandin E2; PD-L1: programmed cell death-ligand 1; CCL/CXCL: chemokine ligand; IL-10: interleukin-10; NK: natural killer; DCs: dendritic cells; TNTs: tunneling nanotubes.

Research has shown that MSCs can secrete bioactive factors, regulating the local immune response directly/indirectly to build a regenerative microenvironment and repair injured tissues. We have summarized the interaction between MSCs and the liver microenvironment in this review (Figure 2).

## 4. MSCs as Hepatocyte-Like Cells

The minimum functional set of hepatocytes includes (i) metabolic and detoxification, (ii) a synthetic function, and (iii) storage. Immunostaining for albumin, cytokeratin-18, or hepatocyte nuclear factor 4 is a recognized indicator of hepatocyte transdifferentiation [36, 42, 43].

In 2002, Schwartz et al. showed that multipotent adult progenitor cells (MAPCs), a subpopulation of MSCs, can express hepatic markers (such as HNF, GATA4, CK18,19, and AFP) and acquire the functional characteristics of hepatocytes (storage and secretion of nutrients and metabolites, phenobarbital-inducible cytochrome P450). They suggested that MSCs might be a promising cell type for managing liver disorders or for use in bioartificial liver devices [44, 45].

A series of subsequent studies also showed that MSCs from sources other than bone marrow could also differentiate into hepatocyte-like cells when cultured with growth factors, cytokine mixtures, resembling the umbilical cord blood-derived MSCs (UCB-MSCs), AT-MSCs, etc. [46–48]. This induced hepatocyte-like cells to express hepatocyte-specific genes and exhibit hepatocyte functions. Lange et al. designed an experiment to compare the differentiation potential of rat BM-MSCs, adult liver cells, and fetal liver cells and examined the influence of the coculture environment on hepatic differentiation [49–51].

Another study in 2006 conducted by Ong et al. showed that cocultured MSCs expressed more hepatic markers and had a greater active metabolic function than the cells only differentiated with growth factors and further verified the assumption that the interactions between the coculture environment and cells may be a significant factor associated with MSC differentiation into hepatocyte-like cells [52]. Several studies have found that, under certain culture conditions, MSCs can express hepatocyte-specific genes and metabolic functions, suggesting that MSCs can transdifferentiate into hepatocyte-like cells *in vitro*. In subsequent years, more evidence was compiled to support this assumption, with many studies confirming the hepatic differentiation potential of MSCs [53–57].

Piryaei et al. found that the population cocultured with nanofibers had more stable hepatic characteristics than those cultured without nanofibers, which may prove the influence of extracellular matrix on MSC differentiation [58].

Although a series of studies found that MSC-derived hepatocytes can home into damaged tissue and function as hepatocytes, the differentiation and homing rates of MSCs are relatively low, as reported in numerous articles. Many reports have noted that the ratio of transdifferentiation into hepatocytes is less than 1% for MSCs after infusion [59]. Following PH, Aurich et al. transplanted MSC-derived hepatocyte-like cells into immunodeficient mice. They found that the population homed into the liver and retained the qualities of hepatocytes (e.g., glycogen storage and ALB production). However, the study also found that homing efficiency was very low, and undifferentiated xenografting MSCs were even excluded from the rat liver [60]. The low rate of homing and differentiation suggested that MSC-mediated therapy might involve other mechanisms aside from direct differentiation [61]. Parekkadan et al. found that the production of MSCs can protect hepatocytes from death and increase hepatocyte survival in cases of hepatic failure [62]. Several experiments have demonstrated a significant reduction in collagen deposition and HSC proliferation and hepatocyte apoptosis, as well as an increase in apoptosis of activated HSCs and hepatocyte proliferation [62–64].

## 5. Mechanical Environment of the Liver and MSCs

In addition to the chelation of endogenous growth factors, the physical properties of the matrix play an essential role in the regulation of MSC activation. Cells can sense physical cues through mechanoreceptors or molecular responses mediated by the cytoskeleton and membrane and then integrate and translate the mechanical signals into biochemical signals controlling the cell fate in a process known as mechanotransduction [65, 66]. The automated environment mainly refers to the forces present at all levels, from the subcellular to the tissue level, the material properties of the surroundings (e.g., the stiffness), the configuration of the extracellular matrix (ECM), and any external mechanical loads applied to the matrix, which play essential roles in embryonic development and tissue repair.

Mechanical forces influence several steps involved in the homing of MSCs, such as vascular adherence and transendothelial migration (TEM). For example, stromal cell-derived factor 1 (SDF-1), the receptor of which (CXCR4) is expressed by MSCs, plays an essential role in MSC recruitment to injured sites [67]. Yuan et al. found that ERK1/2, c-Jun, and p38MAPK proteins are involved in MSC migration under lower shear stress. Furthermore, mechanical strain enhances the expression of CXCR4 on MSCs. The SDF-1/CXCR4 pathway, which is known to be involved in the recruitment of MSCs to injured sites, seems to be augmented by mechanical stimulation [68, 69]. The behavior of MSCs is influenced by paracrine signaling between activated HSCs, portal fibroblasts, and other immune cells.

Baker et al. found that the increase of flat stiffness permitted the amplification of MSCs in vitro but suppressed the spread and proliferation of network structural components. In contrast, the reduced stiffness enabled greater active cellular forces and ligand clustering [70]. Natarajan et al. noticed that matrix stiffness could influence the morphology of primary hepatocytes within physiological ranges. Indeed, hepatocytes cultured on soft (healthy) substrates demonstrated more significant differentiation, were more functional, and lasted longer than the population sophisticated on hard (fiber-like) substrates [70, 71]. In addition to stiffness, elasticity and viscosity also considerably impact MSC activities during culture in vitro. Using a polyacrylamide gel system with a constant compressive modulus and varying loss modulus, Cameron et al. determined that changes to substrate loss modulus can influence the morphology of hMSCs as well as their proliferation and differentiation potential [66]. Bao et al. cultured MSCs via four different methods—single cells (2D), spheroids (3D), 2D+DLS, and 3D+DLS—and discovered that the group which is cultured with 3D+DLS exhibited the higher expression of hepatocyte biological characteristics and specific genes [72]. Subsequent studies support the conclusion that MSCs may sense and respond to the mechanical environment. The automated climate thus regulates MSCs' fate both directly and indirectly.

Hypoxia is a typical pathological inflammatory tissue microenvironment characteristic. Many studies have shown that hypoxic areas appear in the liver after ALI and activated hypoxia-inducible factor-1a (HIF-1a) can be found in liver cells and macrophages of the M1 phenotype. HIF-1a regulates the production of VEGF, PDGF-B, and fibroblast growth factor 2 (FGF-2), promoting fibrosis by inducing HSC activation, proliferation, and collagen production. MSCs exposed to hypoxia have been noticed the secretion of cytokines, such as IL-6, VEGF, and other chemokines [73].

The numerous membrane proteins, cytoskeletal components, and nucleus itself could serve as postulated mechanical sensors in MSCs. Since each cell may express multiple mechanotransducers, it is difficult to discern the primary mechanochemical step. Despite extensive research, the complex interactions between these different actors are still not fully understood.

## 6. The ECM of the Liver and MSCs

The ECM is the protein network forming the general architecture and providing structural support to impart mechanoelastic properties and deliver cues to change the behavior of cells, such as survival, migration, and differentiation [74, 75].

Tissue-specific ECM provides the main favorable regulatory signals in the development and regeneration of several organs, including the lung, kidney, and skin. Advances in organ bioengineering, such as the advent of the acellular liver matrix, have revealed the regulatory role of the liver-extracellular matrix (L-ECM) in the activation and maintenance of liver differentiation in vitr*o* [76–78]. ECM comprises 10% of the volume of a healthy liver [74]. Its accumulation is regarded as the main feature of fibrosis, destroying the liver tissue and the formation of abnormal nodules, leading to cirrhosis. During this progression, many cells, cytokines, and miRNAs are involved. The cycles of apoptosis and regeneration of liver cells exacerbate the process [79].

In 2017, Bi et al. investigated the role of L-ECM in hematogenesis and its mechanism. Using acellular porcine liver homogenate technology, they found that BM-MSCs can express genes of the hepatic lineage and secrete proteins in an L-ECM concentration-dependent manner. In addition, it has been indicated that L-ECM activates a specific type of integrin (ITG) and its downstream signaling pathway. In addition, the cell/ECM interaction is positively affected by the Mn2+ concentration [77].

The chelation of endogenous growth factors is adjusted by ECM protein in response to injury, forming a local inflammatory environment and recruiting immune cells. The cells recruited can also remodel the composition of the ECM in turn [74]. For example, inactive TGF-*β* is associated with a protein complex called the large latency complex (LLC), which anchors to the fibrillary ECM protein through latent-TGF-*β*-binding proteins (LTBPs) [80, 81]. TGF-*β* is released after being activated by either biological or mechanical changes in the complex. Most hepatocytes are susceptible to TGF-*β*. Overexpression of TGF-*β* can induce HSCs to transdifferentiate into myofibroblasts, depositing ECM proteins [82]. TGF-*β* also promotes ECM deposition by amplifying cell death. Furthermore, TGF-*β* released due to the changes in the ECM can drive proinflammatory and inhibitory immune responses [83–85].

Chemokines of ECM act as “homing signals” and participate in the recruitment, migration, and localization of MSCs and other immune cells by producing chemoattractants or immunomodulatory gradients that attract and activate homing immune cells [80, 86, 87]. The chemokine system is a complex system involving mutual effects between chemokines (CCL and CXCL) and specific receptors (CCR and CXCR) [86]. Numerous studies have indicated that MSCs migrate to damaged sites via chemokine receptors, such as CCR1 and CCR4. After migration, they secrete many biologically active and immunosuppressive factors, directly or indirectly involved in immune response regulation [88, 89].

Matrix metalloproteinase (MMP) and its inhibitor TIMP have been found to corrupt proteins and induce reconstruction and turnover in many physiological conditions and illnesses, being considered significant supporters of ECM remodeling [67, 90]. Using a cirrhosis rat model caused by carbon tetrachloride (CCl4), Tanimoto et al. found that BM-MSCs could reverse the fibrotic process induced by remodeling of ECM based on the MMP secretion, a conclusion that is consistent with the findings of Iwamoto and Watanabe [91–93]. Furthermore, MSCs can remodel ECM by suppressing the activation of HSCs and other immune cells, which has been proven in several clinical and preclinical trials [92]. Of note, coculturing MSCs with activated astrocytes resulted in a reduction in collagen deposition and proliferation.

## 7. Crosstalk between MSCs and Liver Cells

### 7.1. Nonparenchymal Cells

#### 7.1.1. LSECs

The liver parenchyma is supported by a vasculature network lined by LSECs, which account for nearly half of liver nonparenchymal cells and play essential roles in pathogen detection, capture, and antigen presentation. LSECs have fenestrations in their healthy state but lose these structures and develop into progressive microvascular dysfunction, causing increased vascular resistance and portal hypertension [79]. This progression occurs before fibrosis and has been observed in several different kinds of liver injury [2, 94].

The increase in portal pressure and decrease in liver volume consequently increase the blood pressure in the hepatic sinusoids, which may stimulate SECs to upregulate the production of uPA [95].

In the first few hours of liver damage, the production of adhesion molecules within LSECs and the release of chemoattractant are increased, including vascular endothelial growth factor (VEGF), downstream stromal cell-derived factor 1 (sdf1), and CXCR7þ, which can result in the recruitment of inflammatory cells to the injury site and start the inflammatory response [96]. Therefore, inflammation and the apoptosis of LSECs are regarded as the initiating events of liver injury.

DeLeve et al. found that it was not mature LSECs driving the process of liver regeneration but BM-derived sinusoidal endothelial progenitor cells, which had been recruited through VEGF-sdf1 signaling. As a receptor of sdf1, CXCR4 is expressed on the surface of resident LSECs and MSCs. VEGF-sdf-1 signaling, after either liver injury or partial hepatectomy, thus recruits MSCs to restore hepatocyte proliferation and completely normalizes liver regeneration in bone marrow-irradiated rats [97].

Chen et al. found that MSC conditioned medium (MSC-CM) can enhance the viability of LSECs with irradiation-related injury and induce the phosphorylation of Akt and ERK *in vitro*. MSC-CM infused before irradiation demonstrated an antiapoptotic effect on LSECs and improved the histopathological characteristics of irradiated injury liver.

In conclusion, MSC-CM can reduce inflammatory cytokine secretion and enhance the production of anti-inflammatory factors [98]. Few studies have explored the crosstalk of LSECs and MSCs [99]. So, further experiments are needed.

#### 7.1.2. HSCs

Several studies have discovered that HSCs will differentiate into different types, such as HPCs, hepatocytes, and BECs, depending on their surroundings, in a process known as epithelial-mesenchymal transition. This process is primarily mediated by canonical Hh signaling. Under pathological conditions, collagen I and fibronectin promote HPC differentiation into hepatocytes and BECs [100]. Kordes et al. noticed that many markers are expressed on pancreatic stellate cells [101]. After HP, these transplanted cells have been found to transdifferentiate into Hnf4*α*+ hepatocytes and panCK+ BECs when cocultured with2AAF/PHX, a hepatocyte proliferation inhibitor. Furthermore, various studies have demonstrated the significant role of HSCs in mass regeneration.

However, there are also many reports concerning the antiproliferative effect of HSCs. While HSCs secrete activated proproliferation cytokines, such as HGF, lFGF, IL-6, NOTCH, and TGF-*β*1, they also produce antiproliferative factors, such as TGF-*β*1. This balance of pro- and antiproliferative cytokines allows HSCs to regulate the restoration of liver mass [102].

Under conditions of inflammation and fibrosis, resident or infiltrating immune cells are activated and secrete a profibrotic factor, which consequently induces HSCs to produce ECM in the liver [103, 104].

MSCs can regulate the proliferation of HSCs and the modulation of ECM, inducing apoptosis of HSCs by secreting HGF, TGF-*β*3, IL-10, and TNF-*α*, among other factors. HGF and TGF-*β*3, in particular, have been found to induce cell cycle arrest of HSCs, leading to growth inhibition, while the other factors inhibit proliferation and ECM synthesis of activated HSCs [105, 106]. MSCs have been proven to restore the albumin level and suppress the transaminase activity and liver fibrosis in rodents [107, 108]. Furthermore, MSCs can increase the MMP expression and decrease the TIMP-1 expression under conditions of liver fibrosis [36, 109].

Regarding their indirect effect, MSCs can regulate the performances of HSCs via the mediation of other immune cells. MSCs are encouraged to migrate to battle-scarred sites by producing inflammatory cytokines, such as IFN-c and IL-1b. In turn, MSCs secrete various mediators (e.g., NO, PGE2, IDO, IL-6, IL-10, and HLA-G), thereby suppressing the proliferation and activating the spread of immune cells [94, 98].

### 7.2. Parenchymal Cells

#### 7.2.1. Hepatocytes

Hepatocytes are responsible for producing most acute-phase proteins and complement components and represent the evolutionarily preserved initial line of defense against pathogens [110, 111]. After the onset of injury or inflammation, liver cells undergo a series of death modes, including apoptosis, necrosis, necroptosis, and autophagy. Production of different types of cell death is released into the local inflammatory environment and circulation, consequently activating nonparenchymal cells (HSCs and KCs) and immune cells once captured, thereby aggravating the damage [112].

MSCs improve the proliferation of hepatocytes through the secretion of proliferative or antiapoptotic cytokines. Antiapoptotic cytokines, such as SDF-1 and VEGF, can effectively scale back the apoptosis of the acquirer via the SDF-1/CXCR-4 axis. In an ALI model, treatment with a conditioned medium from human BM-MSCs resulted in a 90% reduction in cell apoptosis, thus significantly reducing the mortality of rats [113]. Furthermore, MSCs can stimulate the proliferation of hepatocytes and induce liver regeneration by secreting several active cytokines, such as HGF, EGF, and NGF, even during fulminant failure [114–116].

## 8. Crosstalk between MSCs and the Immune Cells

### 8.1. Innate Immunity

NK cells act as vital factors in innate immunity in the liver, playing a significant role in cytolytic activities and the immune response [1, 117]. Using the model of liver injury induced by poly I:C, Qu et al. found that BM-MSCs could inhibit the accumulation and activation of NK cells in the liver. In addition, they also demonstrated that the expression of NK cell type 5 sphingosine 1-phosphate receptor (S1PR5) downregulates the receptor required for NK cells in vivo when cocultured with BM-MSC [118].

MSCs have been proven capable of suppressing the cytolytic activities of resting NK cells by inhibiting the expression of NKp30 and NKG2D, which are involved in the activation of NK cells and targeted cell death [119].

Poggi et al. found that the proliferation of preactivated NK cells cocultured with MSCs reduced IFN-*γ* production and cytotoxicity during coculture with MSCs in vitro. These findings agree with those of Spaggiari. At the same time, it was found that preactivated NK cells had a more substantial effect on MSCs than resting NK cells, and IFN-*γ* was able to protect MSCs from lysis [73, 120].

As a precursor cell rather than an immune cell, MSCs express low MHC I molecules, which NK cell receptors can recognize. When partially incubated with IFN-*γ*, the expression of MHC I on the surface of MSCs increases, protecting the cells from NK cell-mediated cytotoxicity [121].

In conclusion, an IFN-*γ*-rich microenvironment may protect MSCs by inhibiting the NK cell function. In the absence of IFN-*γ*, the balance will eliminate MSCs through activation [122–125].

Macrophages are myeloid-derived cells with several different phenotypes that eliminate pathogens and promote or inhibit inflammation in the liver. In most cases of liver injury, macrophages are highly plastic and heterogeneous [111, 122, 125].

Macrophages originate from either circulating monocytes or are recruited to damaged sites through proinflammatory factors [126–128]. Macrophages are usually classified as the M1 or M2 phenotypes. M1 macrophages play a proinflammatory role during fibrosis, inducing liver injury by secreting superabundant proinflammatory cytokines that activate the inflammatory response and generate reactive oxygen species (ROS). This renders the apoptosis of hepatic cells, thereby aggravating liver damage. M2 macrophages, by contrast, play an anti-inflammatory and profibrotic role in most cases [129]. Macrophages produce C3a and C5a, activate the NF-*κ*B pathway, and induce IL-6 and TNF-*α* synthesis when recruited to injured sites [96].

In the liver injury model induced by ischemia/reperfusion (IR), MSCs reduce hepatocyte damage by enhancing the activity of the Hippo pathway, which can activate NLRP3 and regulate XBP1-mediated NLRP3 and upregulate the translation of YAP and nuclear *β*-catenin subsequently, leading to the polarization of macrophages from M1 to M2 phenotype [130, 131].

There are also two identifiable KC subgroups in the liver under homeostatic conditions: the CD68+ subset, with a phagocytic ability, and the CD11b+ subset, which produces cytokines. Both display a tolerogenic phenotype to maintain immune tolerance and the homeostatic condition of the liver [126]. KCs secrete IL-10 induced by lipopolysaccharide (LPS) and express the T cell inhibitory molecule PD-L1, causing the production of Tregs in a steady state in vivo [1].

Depending on the liver microenvironment, KCs are induced to differentiate into the M1 or M2 phenotype. Murine adipose-derived mesenchymal stem cells (AD-MSCs) were found to upregulate the ratio of M2-like cells by increasing the secretion of IL-10 in mice [111, 132].

Dendritic cells (DCs) play a fundamental role in antigen presentation, inducing tolerogenic responses and activating the immune response, mediated by proinflammatory cytokines and pathogen-associated molecules. Plasmacytoid DCs, a specialized DC population enriched in the liver, are the vital source of IFN*α*, IL-10, and IL-12 [133]. Plasmacytoid dendritic cells (pDCs) produce high levels of type I IFN in response to microbial stimulation and secrete IL-10 and IL-12 in the presence of TLR ligands in vitro. After incubation with MSC, pDCs were shown to upregulate the secretion of the anti-inflammatory cytokine IL-10. Cao et al. found that MSCs can inhibit the maturation of DC in vitro [36]. In addition, the antigen-presentation function of mature DCs had been incubated following coculture with MSCs by downregulating the expression of MHC class II molecules and costimulatory molecules [134].

Neutrophil infiltration, a common occurrence in liver disease, can activate KCs and endothelial cells and upregulate the expression of cellular adhesion molecules, such as ICAM-1 and VCAM-1. Neutrophils can initiate the primary immune process via phagocytosis and the secretion of proinflammatory factors, such as IL-1*β*, TNF, proteolytic enzymes, and oxidative stress, leading to ALI.

Ahn et al. explored the interaction between neutrophils and hUCB-MSCs using a septic mouse model induced by lipopolysaccharide (LPS). They discovered that injected MSCs could adjust the expression of systemic chemokines and cytokines, particularly by increasing the level of IL-10, decreasing mortality, and lessening the severity of sepsis. Furthermore, activated MSCs secrete IL-8 and macrophage migration inhibitory factors (MIF) to recruit neutrophils to engulf MSCs. These gathered neutrophils then contact or exert paracrine effects on other neutrophils, enhancing the function and viability [135, 136].

Under the mediation of chemokines expressed on liver sinusoids, neutrophils migrate to the target organs and accumulate in the liver microvascular system. Subsequently, migration from the sinusoids to the liver parenchyma is achieved by adhesion molecules. It is a complex process involving ATP release, activation of inflammasomes, and foundation of the chemokine gradient (CXCL1, CXCL2, and CXCL8) [137]. The TLR2-mediated signaling pathway plays a fundamental role in neutrophil infiltration. During bacterial infection, neutrophils go through a process called the respiratory burst. MSCs can dampen this respiratory burst and delay the apoptosis of resting mediated by IL-6 and make neutrophils survive [36, 138–140].

### 8.2. Adaptive Immunity

The heterogeneity of T cells concerning their immunological profiles allows them to play both pro- and anti-inflammatory roles in liver disease. The role of T cells in acute and chronic liver injury has been widely studied. In a critical model of acute liver inflammation, the damage was mediated by IFN-*γ* derived from T cells and TNF derived from KCs, leading to the death of hepatocytes, with CD4+ T cells playing a pivotal role. At the same time, Tregs with a high IL-10 secretion was induced, possibly due to the reprogramming of TH1 cells through jagged 1-Notch interaction. In chronic liver diseases, it has been found that CD4+ T cells interact with HSCs, which can be activated by ERK1/2 binding IL-17 directly, leading to further stellate cell activation and the aggravation of fibrosis; in addition, it can changeover fibrosis through the activation of macrophage as well as the production of MMPs.

The interaction between MSCs and adaptive immune cells has been widely studied. The association between MSCs and T cell inhibition was first reported in 1998 [141]. Subsequently, a growing number of studies have shown that MSCs can regulate the activation and function of T cells at several steps in the T-mediated immune response [21, 140, 142].

The ability of MSCs to regulate T cell apoptosis depends on the status of T cell and the microenvironment [143]. Compared with the resting state, activated T cells were shown to be more sensitive to MSC-induced apoptosis, possibly due to the high IDO expression in MSCs. In contrast to mouse MSCs using iNOS, human MSCs used the tryptophan catabolism enzyme IDO to inhibit T cells.

MSCs promote the survival of resting T cells and prevent T cells from incurring activation-induced cell death by downregulating the expression of Fas and FasL on the surface. Akiyama et al. found that MSCs induce autoreactive T cell apoptosis through the FasL-dependent apoptosis pathway, and apoptotic cells also stimulate macrophages to secrete TGF-*β*, which induces Tregs [92]. MSCs can induce T cell hypoxia by inhibiting the expression of cyclin D2 and the proliferation of T cells by releasing NO.

Several factors are involved in this process, including iNOS, IDO, HLA-G, IL-6, IL-10, HO-1, FasL, and PGE2 [80, 87, 144]. CD4+ T cells can be divided into several subsets: proinflammatory subsets, including Th1 and Th17 cells, and anti-inflammatory subsets, including Th2 cells and Tregs. Although MSCs inhibit the proliferation of CD4+ T cells, different subpopulations have different or even opposite responses to MSCs. Others found that human MSCs inhibited Th1 cytokine production and Th17 cell differentiation in a PGE2-dependent manner and increased PGE2 production through the upregulation of PD-1 expression, enhancement of IL-10 secretion, or a CCl2-dependent manner. However, there is substantial evidence that MSCs can promote IL-4 production in Th2 cells, which can suppress abnormal immune responses and promote the repair of tissue [145, 146].

Furthermore, it has been found that MSCs promote CD4+CD25+Foxp3+Treg cells to differentiate into Th1 and Th17 cells and induce CD8+ regulatory T cells to form a coculture system. MSCs can promote Treg differentiation and recruit Tregs to target organs to exert immune-suppressive effects [147].

Interestingly, MSCs reduced the IFN-*γ* production mediated by Th1 cells while increasing the IL-17 production induced by Th17 cells without affecting the IL-10 production by Tregs in mice. Similarly, Svobodova et al. found that mouse MSCs induced naïve T cells to differentiate into anti-inflammatory Tregs and encouraged proinflammatory Th17 cell proliferation. The heterogeneity of these immunological profiles may be due to the secretion of TGF-*β* and IL-6 by MSCs [148, 149].

We previously believed that MSCs mainly regulate the activation and function of adaptive immune cells through paracrine. However, while soluble factors are essential for mediating MSC-based immune suppression, cell-cell contact plays a critical role in MSC-based immunosuppression of T cells.

Mitochondria transplantation from therapeutic cells to target cells has been reported in various tissues and described in numerous studies, resulting in the restoration of the function of mitochondria, increased production of ATP, and rescue of injured cells. This phenomenon has been seen in several diseases [150–153].

Mitochondria transplantation between MSCs and the original cells is primarily mediated by tunneling nanotubes (TNTs) in addition to EVs and cell fusion [153, 154]. TNTs are tubular structures formed by MSCs and the target cells, mediating the trafficking of organelles and biomolecules from one cell to another. They play an essential role in immediate intercellular communication [155]. Mitochondrial trafficking has been extensively researched and is presumed to be an essential mechanism MSCs to repair damage and promote regeneration [156]. Many studies have observed that MSCs can form TNTs with immune cells, altering the injured cells' metabolism and influencing the differentiation of lymphocyte populations via the trafficking of organelles and biomolecules. In coculture of MSCs and proinflammatory Th17 lymphocytes in vitro, TNTs formed within one hour, transferring the mitochondria to lymphoid cells. The transferred mitochondria were subsequently able to do their physiological function. Initially, active Th17 cells express elevated concentrations of IL17 before having their phenotype modified to express FoxP3 and upregulate the production of IL10 while decreasing IL17. Another study found that monocytes in coculture had been induced to alter their metabolism and cytokine expression [157, 158]. Although mitochondrial affect liver cells as high energy need organ and play an important role, there are still not many related articles.

Although the mechanism remains unresolved, a growing number of studies have indicated that mitochondrial translation occurs even under physiological conditions, reporting the transfer of mitochondria from MSCs to immune cells, including 56% in CD4+ cells, 17% in CD8+ cells, and 24% in B cells, while the tissue-specific cells, which can increase the transcription of mRNA related to the activation and cell differentiation of T cells, such as FOXP3, IL2RA, CTLA4, and TGF*β*1, increase the population with highly suppressive CD25+ FoxP3+ [159]. However, little is known about the exchange of organelles between liver cells and MSCs and the role of MSC-derived mitochondria. Generally, MSCs can upregulate the expression of Miro1, the adaptor protein involved in the movement of mitochondria along microtubules, through co-coculturing, the increased expression of TNF*α*-IP2 or antioxidant treatment.

In summary, the above studies mainly focused on the direct regulation of MSCs on T cells. It has been found that MSCs can indirectly promote the production of IL-10 or TGF-*β* by macrophages, induce tolerance among DCs, inhibit the proliferation of T cells, and induce the production of Tregs [160]. Nevertheless, their role in regulating cell function still needs to be further explored.

We still lack awareness of the MSC-mediated regulation of B cells in the liver. Asari et al. found that MSCs suppressed the proliferation of B cells by inducing G0/G1 cell cycle arrest and releasing Blimp-1, a soluble factor necessary for antigen production. Intercellular communication also plays a significant role in MSC-based B cell immunosuppression, which is medicated through PD-1. Controversial results have also been reported regarding the effect of human MSCs on immunoglobulin production. In the extracorporeal system, human MSCs were reported to inhibit the production of IgM, IgG, and IgA of B cells in vitro. However, in another study, MSCs were shown to promote antibody production [161, 162]. The detailed molecular mechanism needs further exploration.

The immunomodulatory function of MSCs shows a high degree of plasticity, which is modulated by the background of the inflammatory microenvironment. MSCs are reportedly effective only when severe inflammation is already underway, and the therapeutic effects observed in the early application of MSCs are poor. Inoue et al. found that MSCs may accelerate graft rejection when combined with CsA to suppress inflammation already underway [161]. All the above studies support our belief that the inflammatory state would affect the immunomodulatory function of MSCs.

## 9. Conclusions

Liver disease is a significant health problem that endangers human health around the world. MSC cell therapy is an exciting treatment method that has begun to be explored in clinical trials and may further break the boundaries of our understanding of the therapeutic possibility and bring about even more significant benefits to patients. Despite several preclinical trials having confirmed the considerable efficacy of MSC therapy in liver diseases, the questionable safety and efficacy still limit its application. A growing number of studies have focused on the anti-inflammatory and immunomodulatory effects of MSCs and strategies to improve the efficiency and safety of MSC treatment. We summarized the interaction between MSCs and the liver microenvironment and expected our findings to function as a reference for improving the safety and efficacy of MSC treatment. Only through further studies will we improve the therapeutic effects of MSC transplantation for liver regeneration to enhance the quality of life and prolong the survival time of patients with liver fibrosis.
